# Acute cervical lymphadenitis and infections of the retropharyngeal and parapharyngeal spaces in children

**DOI:** 10.1186/1472-6815-14-8

**Published:** 2014-09-05

**Authors:** Emilie Georget, Anne Gauthier, Lydia Brugel, Suzanne Verlhac, Natacha Remus, Ralph Epaud, Fouad Madhi

**Affiliations:** 1 Service de Pédiatrie, Centre Hospitalier Intercommunal de Villeneuve-Saint-Georges, 40, allée de la source, 94195 Villeneuve Saint Georges, France; 2 Service d’ORL, Centre Hospitalier Intercommunal de Créteil, 40 avenue de Verdun, 94000 Créteil, France; 3 Service de Radiologie, Centre Hospitalier Intercommunal de Créteil, 40 avenue de Verdun, 94000 Créteil, France; 4 Service de Pédiatrie, Centre Hospitalier Intercommunal de Créteil, 40 avenue de Verdun, 94000 Créteil, France; 5 Inserm U955, Equipe 11, Faculté de Médecine, Université Paris-Est, 94000 Créteil, France

**Keywords:** Acute cervical lymphadenitis, Infection, Retropharyngeal and parapharyngeal space, Children

## Abstract

**Background:**

Acute cervical adenitis can evolve into suppurative cervical lymphadenitis and may sometimes be associated with infection of the retropharyngeal and parapharyngeal spaces (i.e., retropharyngeal and poststyloid parapharyngeal abscesses). This study aimed to describe the clinical presentation of acute cervical lymphadenitis and infections of the retropharyngeal and parapharyngeal spaces in children and examine the management of these conditions.

**Methods:**

This was a retrospective study including children from 3 months to 18 years old who were hospitalized in the Pediatric Department of the Centre-Intercommunal-de-Créteil between January 2003 and May 2010. Selected cases were based on the diagnosis of acute cervical lymphadenitis, suppurative cervical lymphadenitis, or infections of the retropharyngeal or parapharyngeal spaces. Case history, clinical signs, laboratory tests, imaging, treatment and clinical course were collected from patient charts.

**Results:**

We included 75 children (54 males [72%]); 62 (83%) were < 6 years old. Diagnoses were acute cervical lymphadenitis in 43 patients (57%), suppurative cervical lymphadenitis in 13 (17%), retropharyngeal or poststyloid parapharyngeal abscess in 18 (24%) and cervical necrotizing fasciitis in 1 (1%). In total, 72 patients (96%) presented fever and 34 (45%) had torticollis. Suppurative cervical lymphadenitis or abscesses of the retropharyngeal or poststyloid parapharyngeal spaces was significantly higher for children with than without torticollis (52.9% vs. 4.8%, p < 0.001). In all, 21 patients among the 44 > 3 years old (48%) underwent a rapid antigen detection test (RADT) for group A beta-hemolytic *Streptococcus pyogenes*; results for 10 were positive (48%). Contrast-enhanced CT scan of the neck in children with torticollis (n = 31) demonstrated an abscess in 21 (68%). Fine-needle aspiration was performed in 8 patients (11%) and 8 (11%) required surgical drainage. Bacteriology was positive in 8 patients (11%), with a predominance of *Staphylococcus aureus* and *S. pyogenes*. All patients received intravenous antibiotics and the outcome was favorable regardless of surgery. Recurrence was observed in only 1 case among the 34 patients with a follow-up visit after discharge.

**Conclusion:**

Our data suggest that presentation with cervical lymphadenitis associated with fever and torticollis requires evaluation by contrast-enhanced CT scan. Furthermore, abscess drainage should be restricted to the most severely affected patients who do not respond to antibiotic therapy.

## Background

Cervical lymphadenopathy is a frequent occurrence in children and the most common cause of visit to a physician. The origin is usually infectious but can rarely be due to a tumor or congenital malformation. In fact, more than half of the cases of cervical lymphadenopathies in children have a bacterial etiology [1].

Acute cervical adenitis can evolve into suppurative cervical lymphadenitis and may sometimes be associated with infection of the retropharyngeal and parapharyngeal spaces (i.e., retropharyngeal and poststyloid parapharyngeal abscesses). The latter occur rarely, but they require early diagnosis and treatment to avoid possible serious complications such as airway obstruction, epidural abscess, mediastinitis, carotid aneurysm, or cavernous sinus thrombosis [2,3]. Most of the time, these complications occur during nonspecific infections of the nasopharynx or oropharynx that can extend to the retropharyngeal lymph nodes via the lymphatic circulation, thus resulting in retropharyngeal and poststyloid parapharyngeal abscesses [2,3].

We aimed to present our institutional experience in the diagnosis and treatment of acute cervical lymphadenitis and infections of the retropharyngeal and parapharyngeal spaces.

## Methods

This was a retrospective observational study from a university hospital-based pediatric department (Centre Intercommunal de Créteil, Créteil, Val de Marne, France). We included data for all children from 3 months to 18 years old who were hospitalized in the pediatric department between January 2003 and May 2010 with a diagnosis of acute cervical lymphadenitis, suppurative cervical lymphadenitis, infections of the retropharyngeal and parapharyngeal space or cervical necrotizing fasciitis. Our institutional policy is to hospitalize all children when indicated, directly to the general pediatric department, and to manage them in close collaboration with the ear, nose and throat (ENT) specialists.

Case history, biological data, contrast-enhanced CT scan data, treatments and clinical course were retrospectively collected from patient charts. Acute cervical lymphadenitis was defined by clinical signs of fever, neck pain, uni- or bi-lateral latero-cervical adenopathy without fluctuant consistency and by favorable outcome with antibiotic treatment. Suppurative cervical lymphadenitis was defined by acute cervical lymphadenitis with fluctuant consistency and/or fluid collection on cervical ultrasound or CT scan and/or presence of pus after fine-needle aspiration or surgical drainage. Diagnosis of retropharyngeal and parapharyngeal abscesses was based on CT scan with intravenous contrast enhancement demonstrating a fluid collection (i.e., delimited hypodense area with strong contrast enhancement at the periphery), sometimes appearing as a thick rim suggesting the presence of an abscess, and on the specific anatomic location of the collection. Diagnosis of cervical necrotizing fasciitis was based on CT scan demonstrating the presence of necrosis of the subcutaneous soft tissues and superficial fascia, with or without gas development in the fluid collection and on suggestive clinical presentation [4]. The final diagnosis was validated by consensus of a group including 2 ENT specialists and 2 pediatricians, and by a review of the CT scan.

The study, including access to patient record was approved by the institutional review board of Créteil-Intercommunal Hospital.

### Statistical analysis

Data were analyzed by use of Stata SE v11.1 (Stata Corp., College Station, TX, USA). Statistical analyses were primarily descriptive. Chi-square and Fisher tests were used to compare data between groups. The significance level was set at p < 0.05.

## Results and discussion

**Figure 1:**
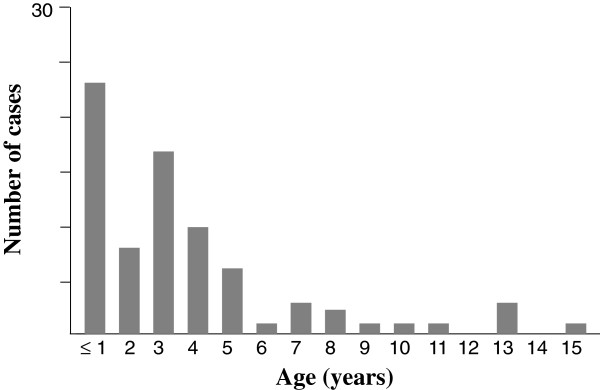
Age of 75 children with acute cervical lymphadenitis and infections of the retropharyngeal and parapharyngeal spaces included in the study.

**Figure 2:**
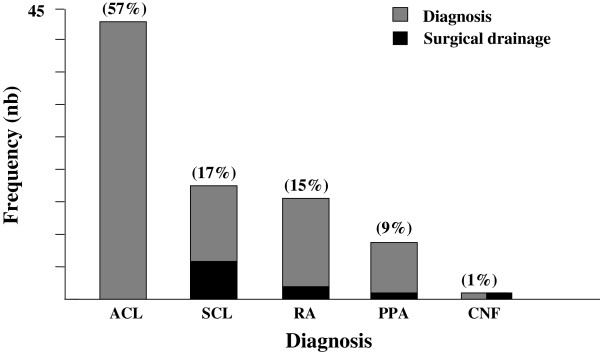
**Frequency of diagnoses and surgical drainage.** ACL: acute cervical lymphadenitis; SCL: suppurative cervical lymphadenitis; RA: retropharyngeal abscess; PPA: poststyloid parapharyngeal abscess; CHF: cervical necrotizing fasciitis. Percentages are given in parentheses.

**Figure 3:**
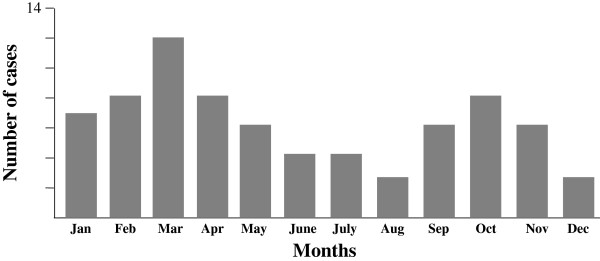
Monthly incidence of acute cervical lymphadenitis and infections of the retropharyngeal and parapharyngeal spaces.

**Figure 4:**
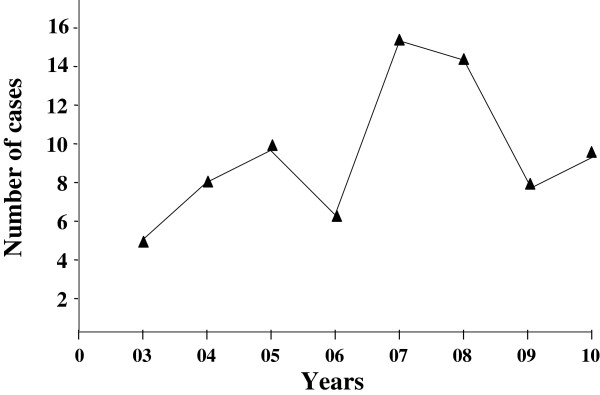
**Annual incidence of acute cervical lymphadenitis and infections of the retropharyngeal and parapharyngeal spaces.** The incidence was calculated for each year from 2003 (03) to 2010 (10).

We included 75 children (54 boys, 21 girls) who were hospitalized during 89 months, and their charts were reviewed. The mean age was 4.2 years and 83% were < 6 years old at the time of diagnosis (Figure 1).Patients underwent ultrasonography (US) or CT scan. A total of 32 patients (43%) underwent cervical US. Multiple abnormal lymph nodes were observed in all cases, and abscess formation was seen in 7 children. These abscesses were diagnosed as cervical necrotizing fasciitis in 1 case and suppurative cervical lymphadenitis in 4, one associated with a retropharyngeal abscess. The last 2 patients with abscess underwent CT to further diagnose the type of abscess (retropharyngeal abscess in 1 case and poststyloid parapharyngeal abscess in the other). In all, 41 children (55%) underwent contrast-enhanced CT; in 31 (76%) images suggested abscess formation. However, for 2 of these patients, the ultimate diagnosis was acute cervical lymphadenitis. The diagnosis for the remaining 29 cases was retropharyngeal abscess in 11, poststyloid parapharyngeal abscess in 7, suppurative cervical lymphadenitis in 10 and cervical necrotizing fasciitis in 1. Thus, the final diagnoses for all 75 patients were acute cervical lymphadenitis in 43 (57%), suppurative cervical lymphadenitis in 13 (17%), retropharyngeal and poststyloid parapharyngeal abscesses in 18 (24%), and cervical necrotizing fasciitis in 1 (1%). No prestyloid parapharyngeal or peritonsillar abscesses were diagnosed during the study period (Figure 2).Although these pathologies were observed during the spring (24%), summer (17%) and autumn (23%), they were more frequent during the winter (36%) (Figure 3). Overall, the number of cases tended to increase each year (Figure 4).

Fever, observed in 96% of children, was the most constant clinical symptom; 65 cases (87%) showed neck swelling and 34 children (45%) presented torticollis (Table 1). Among cases of torticollis, 31 were explored by contrast-enhanced CT, demonstrating that 21 patients (68%) had suppurative cervical lymphadenitis or abscesses of the retropharyngeal or poststyloid parapharyngeal spaces. In contrast, 31 (72%) of the cases of acute cervical lymphadenitis (n = 43) were diagnosed in children who did not have torticollis. Suppurative cervical lymphadenitis or abscesses of the retropharyngeal or poststyloid parapharyngeal spaces was significantly higher for children with than without torticollis (52.9% vs. 4.8%, p < 0.001).

**Table 1:** Clinical and biological characteristics for 75 children with acute cervical lymphadenitis and infections of the retropharyngeal and parapharyngeal spaces

**Diagnosis (n)**	**Fever n (%)**	**Neck swelling n (%)**	**Torticollis n (%)**	**WBC** ≥ **15 000/mm**^**3 **^**n (%)**	**C**-**reactive protein** ≥ **50 mg**/**L n (%)**	**Contrast**-**enhanced CT scan n (%)**	**Microbiological data (n) and method of providing (n)**
**ACL (43)**	42 (97.7)	41 (95.3)	12 (27.9)	26 (60.5)	27 (62.8)	13 (30.2)	*Staphylococcus aureus* (1) Tonsillar swab
**SCL (13)**	12 (92.3)	12 (92.3)	6 (46.2)	9 (69.2)	7 (53.8)	10 (76.9)	*S. aureus* (2) *Streptococcus pyogenes* (1) *Serratia marcescens* (1) *Mycobacterium avium* (1) Surgical drainage (3), fine needle aspiration biopsy (2)
**RA (11)**	10 (90.9)	5 (45.5)	10 (90.9)	8 (72.7)	10 (90.9)	11 (100)	*S. pyogenes* (1) Surgical drainage
**PPA (7)**	7 (100)	6 (85.7)	6 (85.7)	6 (85.7)	6 (85.7)	7 (100)	0
**CHF (1)**	1	1	0	1	1	1	*Streptococcus mitis*, *Prevotella buccae*, *Fusoacterium nucleatum*, *Eikenella corrodens* Surgical drainage

Leukocyte counts were ≥ 15,000/mm^3^ and C-reactive protein level was ≥ 50 mg/L in 5/7 children (71%) with poststyloid parapharyngeal abscess, 8/11 (73%) with retropharyngeal abscess, 18/43 (42%) with acute cervical lymphadenitis and 6/13 (46%) with suppurative cervical lymphadenitis (Table 1). Other biological data were unremarkable.

A total of 21 of the 44 children ≥ 3 years old had throat swabs taken for a rapid antigen detection test (RADT) to detect *Streptococcus pyogenes*; results were positive for 10 patients (48%). Among the children who underwent fine-needle biopsy, drainage or throat culture with a swab of the tonsils (n = 17), the bacteriology was positive in 8 cases (47%), demonstrating infection with *Staphylococcus aureus* (n = 3) or *S. pyogenes* (n = 2). Other bacteria included *Serratia marcescens*, which suggested a diagnosis of chronic granulomatous disease in 1 case, and *Mycobacterium avium* in 1 case in which the clinical course was unfavorable, with fistulization of the suppurative cervical lymphadenitis. Only one sample had more than one bacterium, including *Streptococcus mitis*, *Prevotella buccae*, *Fusobacterium nucleatum*, and *Eikenella corrodens* that were responsible for the 1 case of cervical necrotizing fasciitis (Table 2).

**Table 2:** **Microbiological data after fine**-**needle aspiration biopsy**, **surgical drainage or tonsillar swab**

**Microbiology**	**Number of isolates 8/17** **=** **47%**
**Culture negative**	**8**
**Culture positive**	**9**
*Staphylococcus aureus*	3
*Streptococcus pyogenes*	2
*Mycobacterium avium*	1
*Serratia marcescens*	1
More than one bacterium:	1
- *Streptococcus mitis*	
- *Prevotella buccae*	
- *Fusoacterium nucleatum*	
- *Eikenella corrodens*	

Serological testing for diagnosis of Epstein-Barr virus (EBV), cytomegalovirus (CMV), toxoplasmosis or “cat scratch disease” was rarely performed in our study. EBV serology (n = 28) was positive in 1 case, and CMV serology (n = 18) was positive in 1 case, whereas serology for cat scratch disease (n = 14) demonstrated 1 recent infectious exposure. Fine-needle aspiration biopsy was performed in 8/75 children (11%).

A total of 27 children (36%) had received oral antibiotic therapy as outpatients before hospitalization. During hospitalization, all children received conservative treatment that consisted of intravenous antibiotics. The antibiotics were diverse and included amoxicillin plus clavulanic acid or a third-generation cephalosporin, alone or associated with various drugs such as an aminoglycoside, metronidazole, clindamycin, fosfomycin, vancomycin or rifampicin. In total, 55 children (73%) became afebrile after the third day of antibiotic administration.Treatment by surgical drainage (Figure 2) was deemed necessary for 8 children (11%), with unfavorable clinical, biological and radiological course while on antibiotics. Drainage was performed for 2/11 retropharyngeal abscesses (18%), 1/7 poststyloid parapharyngeal abscess (14%) and 5/13 cases of suppurative cervical lymphadenitis (38%). Four of the 8 patients (50%) who underwent surgical drainage were < 2 years old (n = 23), so 17% of children < 2 years old underwent surgical drainage.

At the time of discharge, all children were disease-free, and follow-up visits were scheduled with an ENT specialist in 44% of the cases and with a pediatrician in 35% of the cases. Only 11% had an appointment to see both an ENT and a pediatrician. Unfortunately, 55% of children were lost to follow-up. For the remaining 45%, all had a favorable outcome, except for 1 who had fistulization and positive bacteriology for *M. avium*.

Cervical lymphadenitis, defined as an acute symptomatic enlargement of the cervical lymph nodes, is a common problem in children of all ages. Most cases of cervical lymphadenitis in children are self-limiting and can safely be monitored for spontaneous resolution over 4 to 6 weeks. Our study in a hospital setting shows that boys appear to be predominantly affected by these infections, even when considering all diagnoses. This finding agrees with the literature, although no clear explanation has been offered [5]. In addition, a large number of children, with acute cervical lymphadenitis, suppurative cervical lymphadenitis or an infection of the retropharyngeal or parapharyngeal spaces, were < 6 years old. This high frequency agrees with the literature [6] and may be explained by the increased number of nasopharyngeal and oropharyngeal infections at this young age. Infections of the nasopharynx and oropharynx can easily spread to the retropharyngeal lymph nodes through the lymphatic circulation, thus resulting in retropharyngeal and poststyloid parapharyngeal abscesses. Cervical lymph nodes progressively atrophy with age, for a decrease in this type of infection with age [2,3,7]. We did not record any prestyloid parapharyngeal or peritonsillar abscesses in our study, perhaps because of the rarity of these infections in children.

The increase in incidence of these infections during January, February and March (36%) may be explained by the greater seasonal frequency of ENT infections in children. Lander *et al*. reported a similar increase during the winter but also during the spring [5]. In our study, the incidence of these infections was lowest in July, August, and September and was intermediate during the spring and autumn months. When considering the incidence of these pathologies over several years, the incidence showed a global tendency to increase between 2003 and May 2010. However, it remained constant when considering only retropharyngeal and poststyloid parapharyngeal abscesses. The literature is mixed in this regard, with some studies reporting an increase in the incidence of neck abscesses [7,8], and others not showing this increase, particularly in the United Kingdom [9].

Clinically, the presence of a torticollis was the most significant symptom suggestive of a diagnosis of abscess. This finding is in agreement with the study by Pelaz *et al*., who also considered torticollis as the most important clinical sign for early diagnosis of abscess [10]. Some studies suggest that CT is particularly sensitive (81%) for diagnosis of retropharyngeal abscess but is not sufficiently specific (57%) [3] to differentiate between a retropharyngeal abscess and a simple cervical lymphadenitis [11]. Thus, we and others suggest that clinical signs should be the prevailing factor in such decision. Although a CT scan alone may not be sufficient for a decision to proceed with surgical drainage, determining the topographical localization of the abscess in relation to other structures is essential when surgical drainage is planned [12]. Courtney *et al*. reported the extensive use of US to explore cervical lymphadenitis (89%), but this test had only a minor impact (10%) on the choice of treatment [13]. We suggest that the use of cervical US be limited to the confirmation of a suppurative lymphadenitis before fine-needle aspiration.

Several studies reported the importance of obtaining samples for bacteriological testing. Others showed that cultures from surgical drainage of retropharyngeal and parapharyngeal abscesses were predominantly positive with *S. aureus* as compared with *S. pyogenes*[6,14]. Coticchia *et al*. reported that in children <1 year old, 79% were infected with *S. aureus* versus 6% with group A Streptococcus. In children ≥ 1 year, 29% were infected with group A Streptococcus versus 16% with *S. aureus*[14]. In our study, only 8 samples were obtained for bacteriology testing, and the results showed a predominance of *S. aureus* and *S. pyogenes*. RADT was performed in 21 children > 3 years old and was positive in almost half of the cases. Since RADT is a non-invasive and easily performed procedure, and despite testing exclusively for *Streptococcus*, the test can contribute to the diagnosis when acute tonsillitis is present on clinical examination.

Niedzielska *et al*. [1] reported that the etiology of cervical lymphadenitis is rarely viral. In our study, serological testing was rarely performed. Gosche *et al*. [15] suggested that cervical lymphadenitis be explored using an algorithm in which serology is considered secondarily in the presence of a symptomatic cervical lymphadenitis with negative cultures and not responding to antibiotic therapy. Hence, serology (for CMV, EBV, toxoplasmosis or cat scratch disease) may be useful as a second line of tests for lymphadenitis cases that are not inflammatory.

In our study, all patients received intravenous antibiotics, and only a small number underwent surgical drainage. With this approach, only one child had a recurrence of suppurative cervical lymphadenitis and fistulization (*M. avium*) during follow-up. Initial empirical treatment should be used discriminately, with consideration of previously described pharmacokinetics and pharmacodynamics data, resistance strains and clinical data of pathogens known to cause infections of the retropharyngeal and parapharyngeal spaces in children (*S. aureus*, *S. pyogenes* and anaerobic bacteria). The initial antibiotic therapy consists in a high dose of parenteral amoxicillin-clavulanic acid, as in recent French recommendations [16]. Using the third-generation cephalosporins with an anti-anaerobic such as metronidazole or clindamycin should be associated. However, cephalosporins should be second-line therapy (in case of penicillin allergy or severe infections) because of potentially selecting ESBL Enterobacteriaceae. Following this work, we have written a document called the “antibio-guide,” which includes treatment protocol for ENT infections.

The optimal treatment for children with retropharyngeal and parapharyngeal abscesses is still a matter of debate. Some groups, including ours, prefer an initial, short period of intravenous antibiotherapy followed by oral antibiotics as soon as the patient is clinically better [3,7,10,17]. Others associate surgical drainage and intravenous antibiotic therapy for retropharyngeal abscesses with diameter > 2 cm on the CT scan [2]. Luu *et al*. showed that despite intravenous antibiotics given in all cases of acute lymphadenitis, 21% still needed surgical drainage. This high number may be explained by 7% of the patients not having cervical lymphadenitis but rather lymphadenitis of the axillary or inguinal regions that are known to be a risk for surgical drainage [6].

Several limitations in our study include its retrospective design and single-centre site. The number of admitted patients was small and limited to the pediatric department, which may explain the absence of peri-tonsillar abscess. Additionally, the timing and indication for surgery were at the discretion of the attending ENT specialist. With regard to antibiotic use, we did not have a specific protocol; the choice of antibiotic was at the discretion of the attending physician. However, the choice of the initial antibiotic was usually reviewed by a multidisciplinary staff, and the causative pathogen was isolated in most patients who underwent surgical drainage. Moreover, we were unable to deeply explore interactions between the season and prevalence of infections because of the small number of patients in each group. Finally, we could have differentiated between acute cervical adenitis and infections of the retropharyngeal and parapharyngeal spaces as in previous studies [1,7,10].

The data reported here provide some input to help improve therapy management in these cases. They also suggest that favourable clinical outcome can be achieved without surgery. However, surgery can be indicated and we believe in close collaboration between ENT specialists and paediatricians to optimize the timing for surgery.

In addition, a follow-up consultation involving the ENT and pediatric specialist needs to be set up at the time of discharge, as our data underscore the difficulty in implementing follow-up and ensuring compliance (55% lost to follow-up). More importantly, we believe that presentation of cervical lymphadenitis associated with fever and torticollis calls for an immediate evaluation of the neck and skull base by contrast-enhanced CT to look for the presence of abscess, evaluate the surrounding vessels, and search for vascular thrombosis.

## Conclusions

Our single-centre experience suggests that contrast-enhanced CT imaging is crucial in determining the cause of acute torticollis in a febrile child. Furthermore, we believe that abscess drainage should be restricted to the most severely affected patients who do not respond to antibiotic therapy.

## Competing interests

The authors report no conflicts of interest. The authors alone are responsible for the content and writing of the paper.

## Authors’ contributions

EG designed and performed the research, collected, performed the statistical analyses and interpreted data, and wrote the manuscript; SV analyzed ultrasonography (US) and CT scan findings; EG, FM, NR, and RE participated in patient care; FM and RE helped write the manuscript. AG and LB participated in the ENT patient care. All authors critically reviewed and approved the manuscript.

## Pre-publication history

The pre-publication history for this paper can be accessed here:

http://www.biomedcentral.com/1472-6815/14/8/prepub
